# Clinical characteristics and immunotherapy response in paraneoplastic neurologic syndrome patients with increased number of high-risk antibodies

**DOI:** 10.3389/fimmu.2024.1520493

**Published:** 2025-01-09

**Authors:** Gong Wang, Mao Chen, Fei Gao, Meng Guo, Maohua Li, Qian He, Jiaojin Jiang, Cheng Huang, Xiaoyan Chen, Rui Xu

**Affiliations:** Department of Neurology, The Second Affiliated Hospital, Army Medical University, Chongqing, China

**Keywords:** PNS, high-risk antibody, number of antibodies, risk factors, immunotherapy response

## Abstract

**Objective:**

To investigate the differences of clinical characteristics and treatment outcomes between paraneoplastic neurologic syndrome (PNS) patients with one high-risk antibody and patients with two high-risk antibodies.

**Methods:**

We retrospectively analyzed the data of 51 PNS patients with high-risk antibody. Clinical data were extracted from the patients’ electronic medical records. Clinical presentations, cerebrospinal fluid (CSF) parameters, radiological characteristics and treatment outcomes between patients with one high-risk antibody and patients with two high-risk antibodies were analyzed.

**Results:**

41 patients with 1 high-risk antibody and 10 patients with 2 high-risk antibodies were enrolled in this study. It was found that psychobehavioral abnormality (OR = 11.327, 95% CI: 1.371 to 93.602, *P* = 0.024), bowel and bladder dysfunction (OR = 23.537, 95% CI: 1.753 to 316.005, *P* = 0.017), and total protein of CSF (OR = 61.556, 95% CI: 2.926 to 1294.974, *P* = 0.008) were risk factors for increased number of high-risk antibodies in PNS. After immunotherapy treatment, Expanded Disability Status Scale (EDSS) scores in PNS patients with 2 high-risk antibodies were higher than that in PNS patients with 1 high-risk antibody (4.8 ± 2.4 vs. 3.0 ± 2.4, *p* = 0.043). EDSS change analysis also revealed that average EDSS score decreased after treatment in PNS with 1 Ab group while increased in PNS with 2 Abs group (*p* = 0.032).

**Conclusions:**

Psychobehavioral abnormality, bowel and bladder dysfunction, and total protein of CSF were three variables associated with increased number of high-risk antibodies in PNS patients, while increased number of high-risk antibodies might indicate a poor immunotherapy response. Our findings might help to understand the association of PNS patients’ clinical features and high-risk antibodies, as well as to guide clinical practice.

## Introduction

Paraneoplastic neurologic syndromes (PNSs) are remote effects of cancer with an immune-mediated pathogenesis (1), which develop in approximately 1 of 300 patients with cancer (2). Antibodies (Abs) are important to guide the search for an underlying tumor, as well as diagnosis of PNS.

According to the frequency of cancer association regardless of their eventual pathogenic effect, PNS antibodies were classified into 3 groups. The first group of antibodies occur very frequently (high-risk, >70%) in patients with an underlying cancer, the second group of antibodies occur in association with cancer in 30%–70% (intermediate-risk) of cases, and the third group of antibodies have a much lower (lower-risk, <30%), or absent, association with cancer. According to updated diagnostic criteria for PNS, high-risk antibodies includes anti-Hu (a.k.a., anti-ANNA-1, associated with small-cell lung cancer, etc.), anti-Ri (a.k.a., anti-ANNA-2, associated with breast cancer, etc.), anti-Yo (a.k.a., anti-PCA-1, associated with ovary and breast cancers), anti-amphiphysin (associated with small-cell lung cancer and breast cancer), anti-Ma2 (associated with testicular cancer, etc.) and anti-Tr (associated with Hodgkin lymphoma) and anti-CRMP-5 (associated with small-cell lung cancer and thymoma), etc. (details of high-risk antibodies were listed in **Supplementary Table S1**). Among those 3 groups of antibodies, high-risk antibodies contribute more points in the PNS-Care Score, a scoring system which is used for the diagnosis of PNS (1). Therefore, high-risk antibody has been studied by the researchers worldwide.

To date, despite the relevant role as biomarkers, high-risk antibodies are believed do not have a direct pathogenic role because they directed against intracellular (e.g., cytoplasmic, nuclear, or synaptic) neuronal antigens, and cytotoxic T cells are thought to exert a pathogenic role (3, 4). Current studies mostly focused on one specific high-risk antibody and its associated PNS, for instance Chatham et al. reported anti-Yo-associated paraneoplastic cerebellar degeneration (5) and Guo et al. reported anti-Ma2 antibody-associated PNS in a pilot study (6). However, it is not rare to see coexistence of two or more autoantibodies in one patient in the clinic. So far, only a few case reports reported some PNS cases with two or more high-risk antibodies, for instance Li et al. reported a PNS case with positive anti-Hu and anti-Yo antibodies (7), and Lockhart et al. reported 2 cases with multiple neural autoantibodies (8). However, these case reports only gave brief descriptions of clinical presentation and response to treatment for these very few patients, therefore, clinical characteristics of PNS patients coexistence of two or more high-risk antibodies is still need to be analyzed to have insight into PNS, as well as to guide clinical practice for PNS patients.

In the presented study, we retrospectively investigated the differences of clinical presentations, cerebrospinal fluid (CSF) parameters, radiological characteristics and treatment outcomes between patients with one high-risk antibody (Ab) and patients with two high-risk Abs, to further understand the association of PNS patients’ clinical features and high-risk antibodies, as well as to guide clinical practice.

## Methods

### Participants

We retrospectively analyzed the data of 51 patients who were included in the database of the Department of Neurology at the Second Affiliated Hospital of Army Medical University from December 2018 to October 2023. The inclusion criteria for patients were as follows: (1) PNS diagnosis was verified according to recently updated criteria (1); (2) Patients were identified with one or two high-risk antibodies which are defined in the updated criteria (1); (3) Patients had received only one of the three kinds of first line treatments (9): (1) intravenous (i.v.) steroids (Methylprednisolone 1g i.v. for 2–5 days followed by gradual tapering; monthly administrations might be required), i.v. high-dose immunoglobulins (0.4 g/Kg/day for 2–5 days; monthly administrations might be required), and plasma-exchange (30-50 ml/Kg/day might be exchanged for 3-5 days; monthly administrations might be required).

The exclusion were (1) Diagnosis of PNS could not be verified; (2) Patients were identified with three or more high-risk antibodies, or coexistence with intermediate-risk antibodies and lower-risk antibodies which were listed in the updated criteria (1); (3) Patients who had received multiple first line immunotherapies, second line immunotherapies or had not receive immunotherapies.

The study was approved by the Medical Ethics Committee of the Second Affiliated Hospital, Army Medical University. The study protocol was performed in accordance with relevant ethical guidelines and regulations for human studies.

### Patient data collection

Demographic data and clinical information, including the age of the patients, sex, BMI, latency at treatment, hospital stay, hospitalization expense, presence of cancer, abnormal of tumor markers, neurological manifestations, brain MRI studies, CSF analysis and Expanded Disability Status Scale (EDSS) scores, were collected by retrospective review of medical records. Data were collected at initial diagnosis before immunotherapy, while for immunotherapy treatment outcome evaluation, data included EDSS, epilepsy, fever, headache and disturbance of consciousness were also collected after immunotherapy.

### Statistical analysis

The data are reported as the mean ± SD for continuous variables and as absolute numbers and percentages for categorical variables. Statistical analysis was performed by using an independent-samples *t* test for continuous variables. Chi-square analysis was used for categorical variables. When chi-square analysis was not appropriate for less frequent occurrences, Fisher’s exact test was performed. Univariate analysis was used to investigate clinical symptoms, radiological characteristics for association with number of high-risk antibodies. Finally, the possible confounding factors were further adjusted using multivariable logistic regression analysis (10) and all the variables with *p* < 0.3 were entered in the regression analysis. Any value expressed as *P* < 0.05 was considered significant. Statistical analyses were conducted using SPSS 18.0.

## Results

### Demographic features

**Figure 1:**
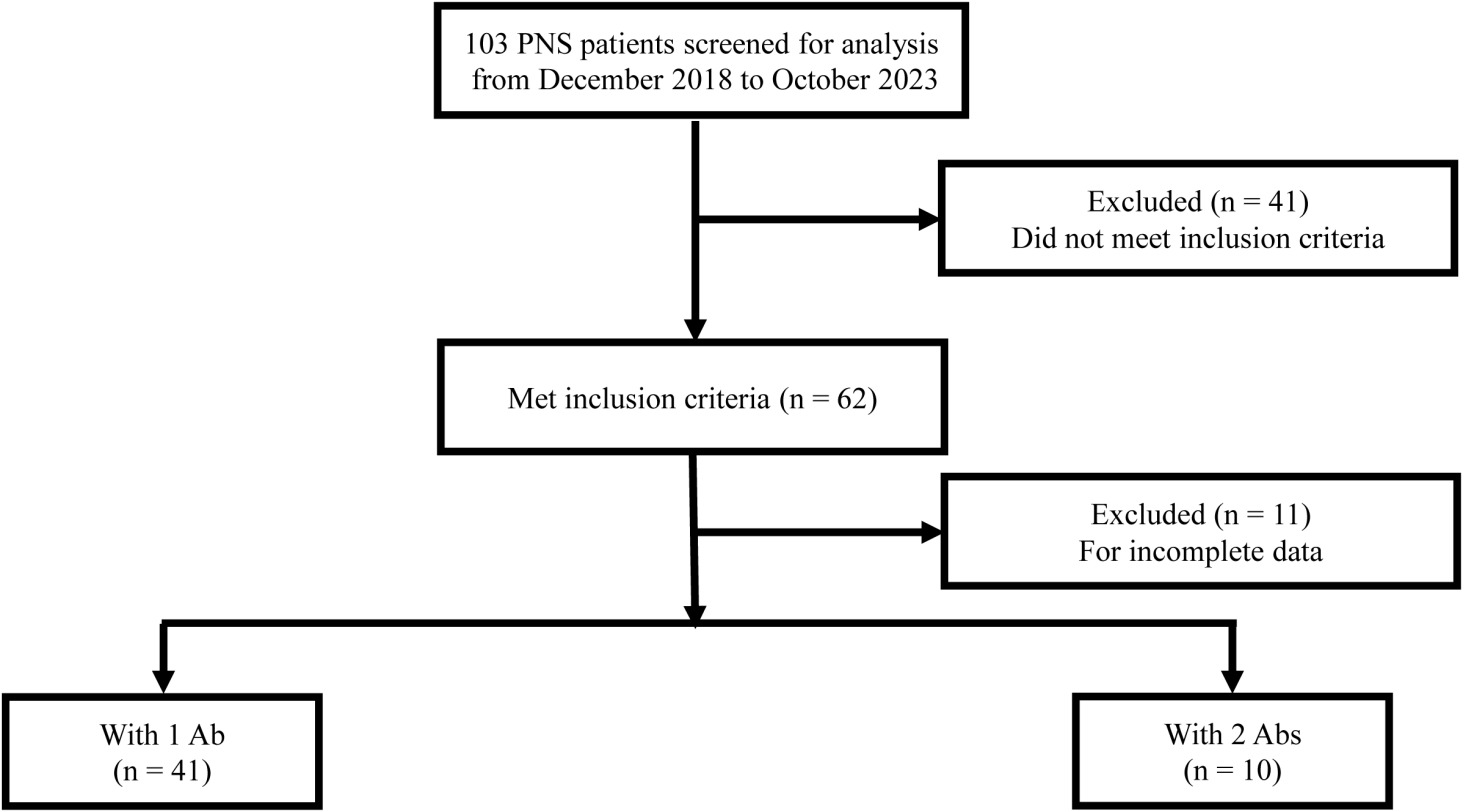
Flow diagram of patient selection.

As shown in **Figure 1**, 51 PNS patients who met inclusion criteria were included in our study, of whom 41 patients with 1 high-risk antibody and 10 patients with 2 high-risk antibodies. The demographic features of patients with 1 high-risk antibody or 2 high-risk antibodies were shown in **Table 1**. Characteristics such as sex distribution, age, latency at treatment, hospital stay, hospitalization expense were similar without significant differences. More importantly, presence of cancer, abnormal of tumor markers were also similar without significant differences in two groups.

**Table 1:** Characteristics of PNS patients with high-risk antibody.

Variables	No. (%) of patients		*P*
	With 1 Ab (n = 41)	With 2 Abs (n = 10)	
**Sex** Male Female	20 (48.8%) 21(51.2%)	4 (40.0%) 6 (60.0%)	0.731
Age (year, mean ± SD)	55.0 ± 15.1	55.0 ± 14.8	0.993
BMI (kg/m^2^, mean ± SD)	23.6 ± 3.8	21.9 ± 4.4	0.216
Latency at treatment (month, mean ± SD)	6.2 ± 10.4	7.4 ± 11.1	0.746
Hospital stay (days, mean ± SD)	13.2 ± 4.5	12.9 ± 5.2	0.857
Hospitalization expense (CNY, mean ± SD)	2.6 ± 2.4	2.0 ± 1.0	0.492
High-risk antibodies involved			
Hu	0	7 (70.0%)	**0.001**
CV2/CRMP5	2 (4.9%)	1 (10.0%)	0.488
SOX1	7 (17.1%)	7 (70.0%)	**0.002**
Yo	10 (24.4%)	3 (30.0%)	0.701
Ri	4 (9.8%)	0	0.573
Tr (DNER)	5 (12.2%)	1 (10.0%)	0.999
Amphiphysin	9 (22.0%)	0	0.176
Ma2 and/or Ma	4 (9.8%)	0	0.573
Presence of cancer	15 (36.6%)	4 (40.0%)	0.999
Abnormal of tumor markers	14 (34.1%)	5 (50.0%)	0.470
First line immunotherapies received			0.898
Intravenous steroids	8 (19.5%)	2 (20.0%)	
High-dose immunoglobulins	20 (48.8%)	4 (40.0%)	
Plasma-exchange	13 (31.7%)	4 (40.0%)	

Bold values were values which were significant different between groups (p < 0.05).

Besides, the presence frequency of two high-risk antibodies, Hu and SOX1, were higher in PNS with 2 Abs group than that in PNS with 1 Ab group. No significant difference of the type of immunotherapy provided to patients was found between one and two high-risk Abs groups (**Table 1**).

### Outcome after immunotherapy treatment

PNS patients involved in this study had received immunotherapy (steroids, plasma exchange or intravenous immunoglobulins). Before immunotherapy treatment received, it was found that EDSS scores in two groups were similar (1 Ab vs. 2 Abs, 3.6 ± 2.2 vs. 4.2 ± 1.6, *p* = 0.453, **Table 2**). While after immunotherapy treatment, EDSS scores in PNS patients with 2 high-risk antibodies were higher than that in PNS patients with 1 high-risk antibody (4.8 ± 2.4 v.s. 3.0 ± 2.4, *p* = 0.043, **Table 2**). Meanwhile, EDSS change analysis further revealed that average EDSS score decreased by 0.6 points after treatment in 1 Ab group, on the contrary, the average score increased by 0.6 points in 2 Abs group (*p* = 0.032, **Table 2**). Other clinical manifestations which are not included in the EDSS score system were also evaluated to reflect the efficacy of immunotherapy in PNS patients with high-risk antibody, and it was found that epilepsy was poor controlled in PNS patients with 2 Abs after immunotherapy (*p* = 0.035, **Table 2**). The results indicated that PNS with 2 Abs might have a poor response to immunotherapy, compared to PNS with 1 Ab.

**Table 2:** Outcome of PNS patients with high-risk antibody after immunotherapy treatment.

Variables		With 1 Ab (n = 41)	With 2 Abs (n = 10)	*P* *(t or χ^2^)*
EDSS				
EDSS Baseline (mean ± SD)		3.6 ± 2.2	4.2 ± 1.6	0.453
**EDSS after immunotherapy** **(mean ± SD)**		**3.0 ± 2.4**	**4.8 ± 2.4**	**0.043**
**EDSS change after immunotherapy** **(mean ± SD)**		**0.6 ± 1.5**	**-0.6 ± 1.8**	**0.032**
Other clinical manifestations		No. (%) of patients		
**Epilepsy**	Before immunotherapy	6 (14.6%)	3 (30%)	0.353
	**After immunotherapy**	**0**	**2 (20%)**	**0.035**
Fever	Before immunotherapy	4 (9.8%)	1 (10%)	0.999
	After immunotherapy	3 (7.3%)	0	0.999
Headache	Before immunotherapy	8 (19.5%)	1 (10%)	0.667
	After immunotherapy	5 (12.2%)	1 (10%)	0.999
Disturbance of consciousness	Before immunotherapy	5 (12.2%)	2 (20%)	0.612
	After immunotherapy	4 (9.8%)	2 (20%)	0.584

Bold values were values which were significant different between groups (p < 0.05).

**Figure 2:**
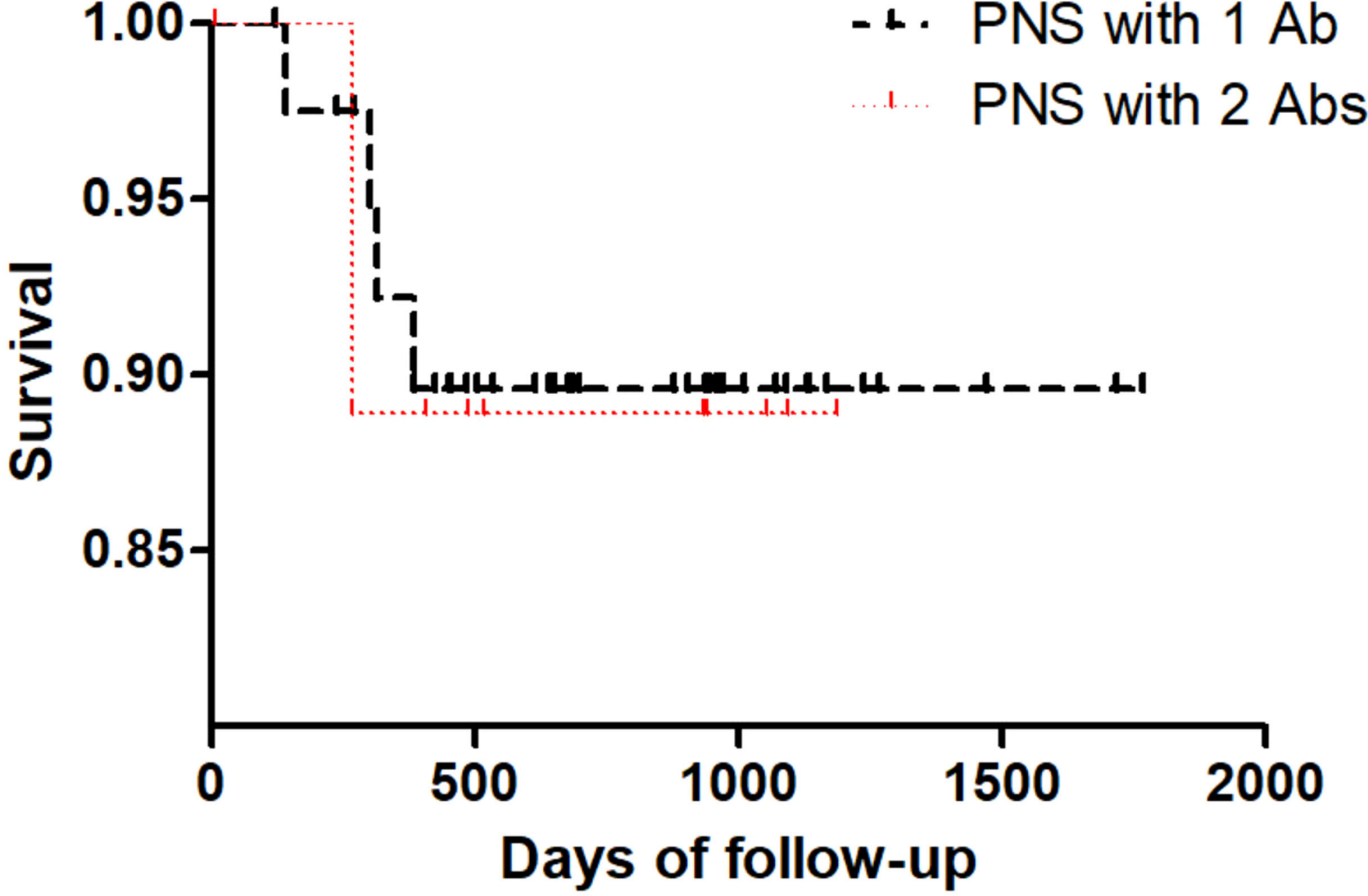
Kaplan–Meier curves of overall survival in PNS patients with 1 antibody and PNS patients with 2 antibodies.

Meanwhile long-term Kaplan–Meier curves are shown in **Figure 2**, overall survival did not significantly differ between PNS with 1 Ab and PNS with 2 Abs (*p* = 0.899).

### Changes in CSF parameters

CSF findings are summarized in **Table 3**. We found that total protein and chlorine in CSF were significantly different between the two groups. The total protein in the 1 Ab and 2 Abs groups were 0.442 ± 0.256 g/L and 0.786± 0.448 g/L, respectively (*P* = 0.041). Meanwhile, the chlorine in the 1 Ab and 2 Abs groups were 127.6 ± 3.4 mmol/L and 124.1 ± 2.8 mmol/L, respectively (*P* = 0.004).

**Table 3:** Analysis of CSF parameters of PNS patients with high-risk antibody.

Variables	With 1 Ab (n = 41)	With 2 Abs (n = 10)	*t*	*P*
Pressure (mmH_2_O)	127.2 ± 46.1	138.5 ± 63.0	-0.0.645	0.522
Cell count	14.7 ± 36.5	13.8 ± 21.1	0.073	0.942
**Total protein (g/L)**	**0.442 ± 0.256**	**0.786± 0.448**	**-2.336**	**0.041**
Glucose (mmol/L)	3.9 ± 0.9	3.6 ± 05	0.817	0.418
**Cl (mmol/L)**	**127.6 ± 3.4**	**124.1 ± 2.8**	**2.991**	**0.004**

CSF, cerebrospinal fluid.

Bold values were values which were significant different between groups (p < 0.05).

### Clinical features and radiological characteristics

As shown in **Table 4**, clinical features were summarized in **Table 4**. It was revealed that two of neurological signs, psychobehavioral abnormality and bowel and bladder dysfunction, were significantly associated with increased number of high-risk antibodies in PNS patients (univariable analysis; psychobehavioral abnormality: OR = 7.286, 95% CI: 1.619 to 32.787, *P* = 0.01; bowel and bladder dysfunction: OR = 8.357, 95% CI: 1.175 to 59.434, *P* = 0.034). While other symptoms or signs like cognitive disorder, memory deterioration and lalopathy were not found to be associated with increased number of high-risk antibodies.

**Table 4:** Univariate logistic-regression analysis to investigate clinical symptoms for association with number of high-risk antibodies in PNS patients.

Clinical Symptoms	OR	95%CI for OR	*P*
**Psychobehavioral abnormality**	**7.286**	**1.619 to 32.787**	**0.010**
**Bowel and bladder dysfunction**	**8.357**	**1.175 to 59.434**	**0.034**
Headache	0.458	0.050 to 4.160	0.488
Fever	1.028	0.102 to 10.346	0.981
Cognitive disorder	2.417	0.590 to 9.902	0.220
Memory deterioration	2.750	0.625 to 12.108	0.181
Lalopathy	0.239	0.027 to 2.092	0.196
Dysphagia	1.800	0.295 to 10.998	0.524
Epilepsy	2.500	0.502 to 12.457	0.263
Status epilepticus	4.444	0.253 to 77.963	0.307
Disturbance of consciousness	1.800	0.295 to 10.998	0.524
Decreased myodynamia	0.920	0.191 to 4.432	0.917
Abnormal sensation^#^	0.984	0.173 to 5.595	0.986
Diplopia	2.312	0.359 to 14.877	0.377
Gait abnormality	1.031	0.183 to 5.825	0.972

Dependent variable: number of high-risk antibodies (1 or 2).

Abnormal sensation^#^: including hyperesthesia, paresthesia, pain, hypesthesia, anesthesia, deep sensation abnormal.

Bold values were values which were significant different between groups (p < 0.05).

As shown in **Supplementary Table S2**, univariable analysis was used to investigate radiological characteristics for association with number of high-risk antibodies. It was found that radiological characteristics like abnormal in brain or/and spinal cord MRI, number of MRI lesions, and gadolinium enhancement were not associated with increased number of high-risk antibodies.

### Psychobehavioral abnormality, bowel and bladder dysfunction, and total protein of CSF were three variables associated with increased number of high-risk antibodies

**Figure 3:**
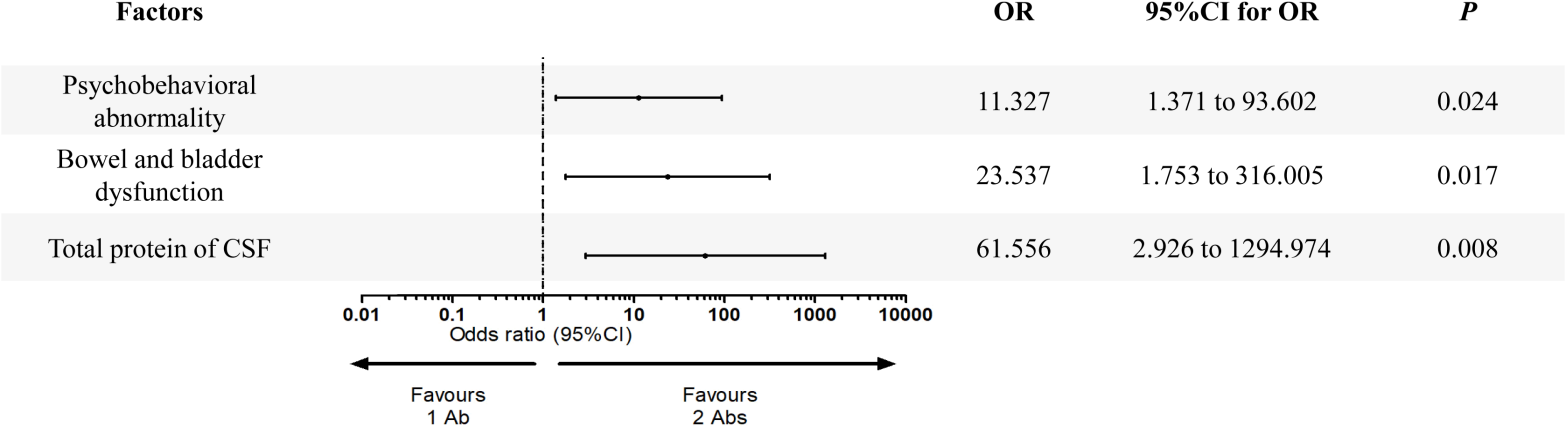
Multivariate logistic regression analysis of variables predicting the number of high-risk antibodies in PNS patients.

Twelve potential variables, including *Psychobehavioral abnormality, bowel and bladder dysfunction, total protein of CSF, chlorine of CSF, cognitive disorder, memory deterioration, lalopathy, epilepsy, BMI, number of MRI lesions, glucose of CSF* and *abnormal of tumor markers* were screened from the statistical analysis mentioned above and entered in the multivariate logistic regression analysis. As shown in **Figure 3**, three variables were confirmed to be associated with increased number of high-risk antibodies, i.e., psychobehavioral abnormality (OR = 11.327, 95% CI: 1.371 to 93.602, *P* = 0.024), bowel and bladder dysfunction (OR = 23.537, 95% CI: 1.753 to 316.005, *P* = 0.017), and total protein of CSF (OR = 61.556, 95% CI: 2.926 to 1294.974, *P* = 0.008).

## Discussion

Clinically, PNS should be suspected in patients with subacute, progressive neurologic symptoms and existing or high risk for malignancy. There are two main classes of autoantibodies, intracellular and cell-surface/synaptic (11). Instead of playing a direct pathogenic role in mediating disease, intracellular antibodies (high-risk antibodies) presence is often used as a marker of disease. However, further investigation of intracellular antibodies’ pathophysiology and its contribution to the neurologic disease process is needed (11). As we found that it was not rare to see coexistence of two or more high-risk autoantibodies in one patient in the clinic, we therefore retrospectively investigated the association of PNS patients’ characteristics and number of high-risk antibodies in this study.

Our study first investigated the association of malignancy and number of high-risk antibodies. Although it was reported the presence of even one high-risk antibody had a strong association with malignancy (8, 12), and in some cases more than one CNS autoantibody (not high-risk antibody) raised the likelihood of a malignancy (13–15), in our study we did not find a stronger association with malignancy when more than one high-risk antibody presented. However, it should be noted that tumor diagnosis may be difficult even in definite paraneoplastic syndromes, detailed investigation like whole body FDG-PET is still recommended (16, 17), and even be repeated at intervals of 4–6 months if no cancer is found on the original investigations (18).

We then investigated the association of PNS patients’ immunotherapy treatment outcome and number of high-risk antibodies. In larger cohorts, outcome often depend on which specific antibody is present, to our knowledge, the relationship between number of high-risk antibodies and immunotherapy treatment response has seldom been discussed. Generally, patients with antibodies against cell surface proteins (i.e. intermediate-risk antibodies or low-risk antibodies) benefit more from immunotherapy (9) than those with antibodies against intracellular targets (high-risk antibodies), however, this can vary (19–21). For example, anti-Hu associated disease may be poorly responsive whereas Ma2 encephalitis may be well responsive with good outcomes (19). In our research, we find that PNS with 2 Abs might have a poor response to first line therapies (steroids, plasma exchange or intravenous immunoglobulins), compared to PNS with 1 Ab. The result may guide to predict outcome when we find a patient with overlapping high-risk antibodies in clinic.

We also investigated the association of PNS patients’ clinical features and number of high-risk antibodies to help to further understand the role of high-risk antibodies in PNS. It was revealed that two CSF parameters (total protein of CSF, chlorine of CSF) and two clinical symptoms (psychobehavioral abnormality, bowel and bladder dysfunction), had significant changes when PNS with 1 Ab group compared with PNS with 2 Abs group. After adjusted for confounding factors, psychobehavioral abnormality, bowel and bladder dysfunction and total protein of CSF were confirmed to be risk factors for increased number of high-risk antibodies (2 Abs). These findings were interesting and might help to predict number of high-risk antibodies in PNS in the clinic, and further to predict treatment response as mentioned above.

In conclusion, in this study we found that psychobehavioral abnormality, bowel and bladder dysfunction and total protein of CSF were risk factors for increased number of high-risk antibodies in PNS, and increased number of high-risk antibodies might indicate a poor immunotherapy response,. Our findings might help to understand the association of PNS patients’ clinical features and high-risk antibodies, as well as to guide clinical practice. This study has a few limitations. First, this was a single-center, retrospective study and might introduce a systematic selection bias. Second, the sample size for PNS with 2 Abs group was relatively small. A multicenter, prospective and controlled trial is still needed in the future to confirm our results.

## Data Availability

The original contributions presented in the study are included in the article/**Supplementary Material**. Further inquiries can be directed to the corresponding authors.
