# Supplementary material for: Clinical characteristics and immunotherapy response in paraneoplastic neurologic syndrome patients with increased number of high-risk antibodies
Source: Front Immunol. 2025 Jan 9;15:1520493. doi: 10.3389/fimmu.2024.1520493 (PMC11754214; doi:10.3389/fimmu.2024.1520493)
Supplement: Supplementary file 1 [file Table1.docx]

***Supplementary materials***

**Clinical characteristics and immunotherapy response in paraneoplastic neurologic syndrome patients with increased number of high-risk antibodies**

Gong Wang ^1,#^, Mao Chen ^1,#^, Fei Gao ^1^, Meng Guo ^1^, Maohua Li ^1^, Qian He ^1^, Jiaojin Jiang^1^, Cheng Huang ^1*^, Xiaoyan Chen ^1,*^, Rui Xu ^1,*^

^1^ Department of Neurology, The Second Affiliated Hospital, Army Medical University, Chongqing 400037, China.

^#^ These authors contributed equally to this work.

*** Correspondence:**

Rui Xu: xurui007@tmmu.edu.cn, Tel: 023-68774213;

Xiaoyan Chen: cq1997@163.com;

Chen Huang: hwangcheng1992@tmmu.edu.cn, Tel: 023-68774413.

**Table S1. High-risk antibodies according to updated diagnostic criteria for paraneoplastic neurologic syndromes ^1^**

| **Antibody (alternative name)** | **Neurologic phenotypes** | **Frequency of cancer** | **Usual tumors** |
| --- | --- | --- | --- |
| Hu (ANNA-1) | SNN, chronic gastrointestinal pseudo-obstruction, EM, and LE | 85 % | SCLC >> NSCLC, other neuroendocrine tumors, and neuroblastoma |
| CV2/CRMP5 | EM and SNN | >80 % | SCLC and thymoma |
| SOX1 | LEMS with and without rapidly progressive cerebellar syndrome | >90 % | SCLC |
| PCA2 (MAP1B) | Sensorimotor neuropathy, rapidly progressive cerebellar syndrome, and EM | 80 % | SCLC, NSCLC, and breast cancer |
| Amphiphysin | Polyradiculoneuropathy, SNN, EM, SPS | 80 % | SCLC and breast cancer |
| Ri (ANNA-2) | Brainstem/cerebellar syndrome, OMS | >70 % | Breast > lung (SCLC and NSCLC) |
| Yo (PCA-1) | Rapidly progressive cerebellar syndrome | >90 % | Ovary and breast cancers |
| Ma2 and/or Ma | LE, diencephalitis, and brainstem encephalitis | >75 % | Testicular cancer and NSCLC |
| Tr (DNER) | Rapidly progressive cerebellar syndrome | 90 % | Hodgkin lymphoma |
| KLHL11 | Brainstem/cerebellar syndrome | 80 % | Testicular cancer |

Abbreviations: ANNA = antineuronal nuclear antibody; CRMP5 = collapsin response-mediator protein 5; DNER = delta/notch-like epidermal growth factor–related receptor; EM = encephalomyelitis; KLHL11 = Kelch-like protein 11; LE = limbic encephalitis; LEMS = Lambert-Eaton myasthenic syndrome; MAP1B = microtubule-associated protein 1B; NSCLC = non–small-cell lung cancer; OMS = opsoclonus-myoclonus syndrome; PCA = Purkinje cell antibody; SCLC = small-cell lung cancer; SNN = sensory neuronopathy; SPS = stiff-person syndrome.

**Reference**

^1^ Graus, F., et al., Updated Diagnostic Criteria for Paraneoplastic Neurologic Syndromes. Neurol Neuroimmunol Neuroinflamm, 2021. 8(4).

**Table S2.** **Univariate logistic-regression analysis to investigate radiological characteristics for association with number of high-risk antibodies in PNS patients**

| **Factors** | **OR** | **95%CI for OR** | ***P*** |
| --- | --- | --- | --- |
| Abnormal in Brain MRI | 0.482 | 0.090 to 2.583 | 0.394 |
| Abnormal in Spinal Cord MRI | 0.800 | 0.083 to 7.727 | 0.847 |
| Abnormal in MRI (Brain + Spinal Cord) | 0.605 | 0.137 to 2.680 | 0.508 |
| Number of MRI lesions (Brain + Spinal Cord) | 0.559 | 0.189 to 1.649 | 0.292 |
| Gadolinium enhancement | 0.540 | 0.059 to 4.971 | 0.586 |

Dependent variable: number of high-risk antibodies (1 or 2).
